# Determinants of synchronous and metachronous metastatic breast cancer (2000–2024)–an analysis based on data from German cancer registries

**DOI:** 10.1007/s00432-026-06631-5

**Published:** 2026-11-04

**Authors:** M. E. Lacruz, A. Bedir, I. Wittenberg, S. Thies, C. Schneider, F. Reinwald, W. Hoffmann, S. Faißt, B. Franke, K. Kleihues, S. R. Zeissig, M. Klinkhammer-Schalke, M. Gerken, E. C. Sturm-Inwald, A. Ignatov

**Affiliations:** 1 Cancer Registry Saxony-Anhalt, Magdeburg, Germany; 2 Clinical-Epidemiological Cancer Registry Brandenburg-Berlin, Cottbus, Germany; 3 Cancer Registry of Rhineland-Palatinate in the Institute for Digital Health Data, Mainz, Germany; 4 Cancer Registry Mecklenburg-Western Pomerania, Institute for Community Medicine, University Medicine Greifswald, Greifswald, Germany; 5 Institute for Health Services Research at the Oncology Center Stuttgart E.V., Stuttgart, Germany; 6 Association of German Tumor Centers (ADT), Network for Care, Quality and Research in Oncology, Berlin, Germany; 7 Institute of Clinical Epidemiology and Biometry, Julius Maximilian University of Würzburg, Würzburg, Germany; 8 Bavarian Cancer Registry-Regional Center Würzburg, Bavarian Health and Food Safety Authority, Würzburg, Germany; 9 Tumor Center Regensburg, Center for Quality Management and Health Care Research of the University, Regensburg, Germany; 10 Department of Epidemiology and Preventive Medicine, University of Regensburg, Regensburg, Germany; 11 Bavarian Cancer Registry, Bavarian Health and Food Safety Authority, Regensburg, Germany; 12 University Hospital for Obstetrics and Gynecology, Medical Faculty, Otto-Von-Guericke University, Magdeburg, Germany

**Keywords:** Breast cancer, Molecular subtype, Synchronous metastasis, Metachronous metastasis, Metastatic pattern, Cancer registry

## Abstract

**Purpose:**

To investigate the association of molecular breast cancer subtypes with the timing and organ-specific patterns of metastatic disease.

**Methods:**

We analyzed data from nine German regional cancer registries, including 172 512 women diagnosed with invasive breast cancer between January 2000 and March 2024. Patients were classified as having no metastases, synchronous metastases (within three months of diagnosis), or metachronous metastases. Factors associated with synchronous metastases were assessed using multivariable binary logistic regression adjusted for confounders. Time to first metachronous metastasis was analyzed using multivariable Cox regression. Timing and organ-specific metastatic patterns were compared across subtypes.

**Results:**

Molecular subtypes were associated with both synchronous and metachronous metastatic disease. Compared with HR+/HER2− tumors, HR−/HER2+ tumors had the highest odds of synchronous metastasis (adjusted OR [aOR] 1.69, 95%CI 1.57 – 1.83), whereas HR−/HER2− tumors showed no increased odds (aOR 0.96, 95%CI 0.90 – 1.02). In contrast, HR−/HER2− tumors had the highest risk of metachronous metastases (adjusted HR [aHR] 1.48, 95%CI 1.41 – 1.55), while HR+/HER2+ tumors showed a lower risk than HR+/HER2− tumors (aHR 0.88, 95%CI 0.84–0.93). Cumulative incidence increased between 5 and 10 years after diagnosis, with late risk among HR-positive tumors. Metastatic patterns differed by subtype, with HR+/HER2− tumors showing predominantly bone metastases, and HR−/HER2− tumors exhibiting more frequent visceral and central nervous system (CNS) involvement.

**Conclusion:**

Molecular subtypes influence the timing and distribution of metastatic breast cancer. HER2-positive tumors are associated with synchronous metastatic presentation, whereas HR−/HER2− tumors showed the highest risk of metachronous metastasis. These findings support subtype-specific risk assessment and surveillance strategies.

## Introduction

Breast cancer is the most frequently diagnosed malignancy among women worldwide and remains a leading cause of cancer-related mortality (Sung et al. 2021). Most breast cancer deaths are attributable to metastatic disease rather than the primary tumor itself, highlighting metastases as the critical determinant of long-term prognosis. Despite substantial advances in screening, systemic therapies, personalized treatment strategies and aftercare, metastatic breast cancer remains largely incurable (Riggio et al. 2021; Mani et al. 2024; Sundén et al. 2026).

Metastatic breast cancer comprises two clinically distinct entities. Synchronous (de novo) metastatic disease is characterized by distant metastases detected at or shortly after the initial breast cancer diagnosis, whereas metachronous metastatic disease develops following an interval of apparently localized disease. Increasing evidence suggests that these entities differ with respect to tumor biology, metastatic patterns, treatment exposure, and survival outcomes. Metachronous metastases may arise from dormant disseminated tumor cells that evade initial treatment and become clinically apparent years after diagnosis, whereas synchronous metastases may reflect an intrinsically aggressive metastatic phenotype (Riggio et al. 2021; Slotman et al. 2026; Malmgren et al. 2018).

Several studies have investigated risk factors and outcomes of metastatic breast cancer. However, most were derived from clinical cohorts, single-center experiences, or selected metastatic populations. Furthermore, population-based analyses that simultaneously examine synchronous and metachronous metastases across contemporary breast cancer subtypes remain scarce. Existing registry studies have frequently focused on survival after metastatic diagnosis rather than on determinants of metastatic spread throughout the disease course (Slotman et al. 2026; Malmgren et al. 2018; Lord et al. 2022).

Therefore, the aim of the present study was to investigate factors associated with synchronous and metachronous metastases and to characterize patterns of metastatic spread in a large population-based cohort of women with invasive breast cancer. Population-based cancer registry data provide a comprehensive representation of women with invasive breast cancer treated in routine clinical practice and enable the investigation of metastatic patterns beyond the selected populations typically represented in clinical studies (Katalinic et al. 2023). Specifically, we examined factors associated with the presence of synchronous metastases at diagnosis, the development of metachronous metastases during follow-up, and patterns of metastatic spread according to clinicopathological characteristics and molecular breast cancer subtypes.

## Methods

### Data source and study population

This population-based cohort study used data from nine participating German cancer registries contributing to the Association of German Tumor Centres (ADT) call for the National Conference on Quality in Oncology 2026, including regional population-based cancer registries and hospital-based registries located at tumor centers. Women aged 18 years or older diagnosed with invasive breast cancer between January 1, 2000 and March 31, 2024 were eligible.

Patients were identified by the ICD-10-GM (International Classification of Diseases – 10th Revision – German Modification) diagnosis code C50 (malignant neoplasm of breast) and International Classification of Diseases for Oncology, Third Edition (ICD-O-3) morphology codes 8500/3, 8520/3, and 8522/3. The analysis was restricted to these three common invasive breast cancer morphology codes to focus on the major invasive epithelial breast cancer subtypes and to exclude rare histological entities with substantially different biological characteristics and clinical behavior.

Potentially overlapping records across registries were avoided by linkage according to region of the registry and the patients place of residence, thereby preventing duplicate registration of the same cancer case. Patients with multiple primary breast cancers were excluded because the available data will not always allow reliable attribution of a subsequent metastatic disease to a single primary tumor. To ensure complete and comparable follow-up across registries, data were administratively censored on September 30, 2024. Patients with missing hormone receptor (HR) or Human Epidermal Growth Factor Receptor 2 (HER2) status were excluded. The final study cohort comprised 172 512 women.

### Definition of metastatic disease

Information on distant metastases was obtained from registry records documenting metastatic disease at diagnosis, recurrence, or disease progression. Registry records were retrospectively linked with administrative records to identify deaths during follow-up. No separate case-by-case clinical validation of all recurrence records was performed; completeness depended on the available registry and administrative documentation.

For this study, metastases detected within 92 days of primary breast cancer diagnosis were classified as synchronous. The 92 days cutoff is used in all German cancer registries according to the main reference manual and European standards (Stegmaier et al. 2019; Gavin et al. 2015; Tyczynski et al. 2003). This interval is intended to offer enough time for initial staging to be finished. To reduce misclassification of early disease progression as de novo metastatic disease, metastases detected within this period were considered synchronous only if no systemic treatment had been initiated before metastatic disease was documented; otherwise, they were classified as metachronous. The available data did not permit a reliable case-level distinction between delayed completion of staging and disease progression after non-metastatic baseline in every case; the treatment-timing rule was therefore used as a prespecified registry-based approach to reduce the latter misclassification. Metastases first documented more than 92 days after diagnosis were classified as metachronous. Patients without documented distant metastases at diagnosis or during follow-up were classified as non-metastatic.

To assess the potential impact of the treatment-timing criterion, patients with metastases documented within 92 days of diagnosis were evaluated according to whether systemic treatment had already been initiated; only 83 of 11 373 patients (< 1%) were reclassified as non-synchronous, with similarly low proportions across molecular subtypes and calendar periods (Supplementary Table S1).

### Variables

Patient and tumor characteristics included age at diagnosis, year of diagnosis, tumor stage (tumor size [T] and lymph nodal involvement [N]), histological grade, molecular subtype, treatment variables, and registry source. Registry source indicated the cancer registry from which the information was obtained. Breast cancer subtypes were defined by HR and HER2 status as HR+/HER2−, HR+/HER2+, HR−/HER2+, and HR−/HER2− (triple-negative breast cancer [TNBC]).

Tumor stage was derived from clinical and pathological TNM classifications according to Wittekind (Wittekind 2017). Pathological staging was used when available, except for patients receiving neoadjuvant therapy, for whom clinical stage at diagnosis was used to avoid treatment-related downstaging. If pathological stage was unavailable, clinical stage was used. Histological grade was categorized as grade 1–3 or unknown.

Primary treatment was defined as treatment received within six months after the initial breast cancer diagnosis. For patients who subsequently developed metachronous metastases, only treatments administered before the diagnosis of metastatic disease were considered. Treatment variables included surgery, chemotherapy, endocrine therapy, and radiotherapy. Surgery was categorized as breast-conserving surgery/mastectomy, other surgery, or no surgery. Endocrine therapy was evaluated only within biologically eligible subgroups.

### Follow-up and outcome definition

For analyses of time to metachronous metastases, the eligible population comprised patients without synchronous metastases according to the above definition, including patients whose metastases were detected within 92 days but only after systemic treatment had already been initiated. Time zero was the date of primary breast cancer diagnosis. Follow-up continued until the first occurrence of metachronous distant metastases, death, or administrative censoring (30 September 2024), whichever occurred first.

The primary time-to-event outcome was the occurrence of metachronous metastatic disease.

### Statistical analysis

Baseline characteristics were summarized according to metastatic status (synchronous, metachronous, and non-metastatic disease).

Associations between patient and tumor characteristics and synchronous metastatic presentation were evaluated using multivariable binary logistic regression. Results are reported as odds ratios (ORs) with 95% confidence intervals (CIs). The synchronous model was intended to estimate overall associations with metastatic presentation at diagnosis and therefore did not adjust for T or N stage, which represent measures of disease extent at the time of diagnosis and are closely related to the metastatic presentation itself. Models included age at diagnosis, histological grade, calendar period (2000–2009; 2010–2016; 2017-March 2024), and registry source. Calendar period was included to account for temporal changes in the systemic treatment landscape, whereas registry source was included to account for differences in documentation practices, data collection procedures, and registry-specific definitions.

Among patients without synchronous metastases, cumulative incidence of metachronous metastases was estimated using Kaplan–Meier methods (1 − survival probability) and stratified by molecular subtype. The median follow-up time was estimated using the reverse Kaplan–Meier method.

Factors associated with metachronous metastases were assessed using multivariable Cox proportional hazards model. Hazard ratios (HRs) and 95% CIs were estimated after adjustment for age, calendar period, T stage, N stage, histological grade, molecular subtype, and registry source. Registry source was included as a term to account for between registry heterogeneity. The proportional hazards assumption was assessed using graphical diagnostics and inspection of Schoenfeld residual plots.

To characterize metastatic dissemination patterns, the first documented metastatic site was classified as bone, visceral, CNS, or other/multiple sites. Visceral metastases included internal organs such as lung, liver, peritoneum, or pancreas. The category other comprised metastatic sites not classified as bone, visceral or CNS, including for example skin, lymph nodes, bone marrow or unspecified sites. Distributions were summarized descriptively according to molecular subtype and timing of metastatic presentation (synchronous versus metachronous), with the denominator defined as the number of organ-specific metastases in each subtype.

All analyses were performed using R version 4.3.2 (R Foundation for Statistical Computing, Vienna, Austria).

## Results

### Cohort description

**Fig. 1:**
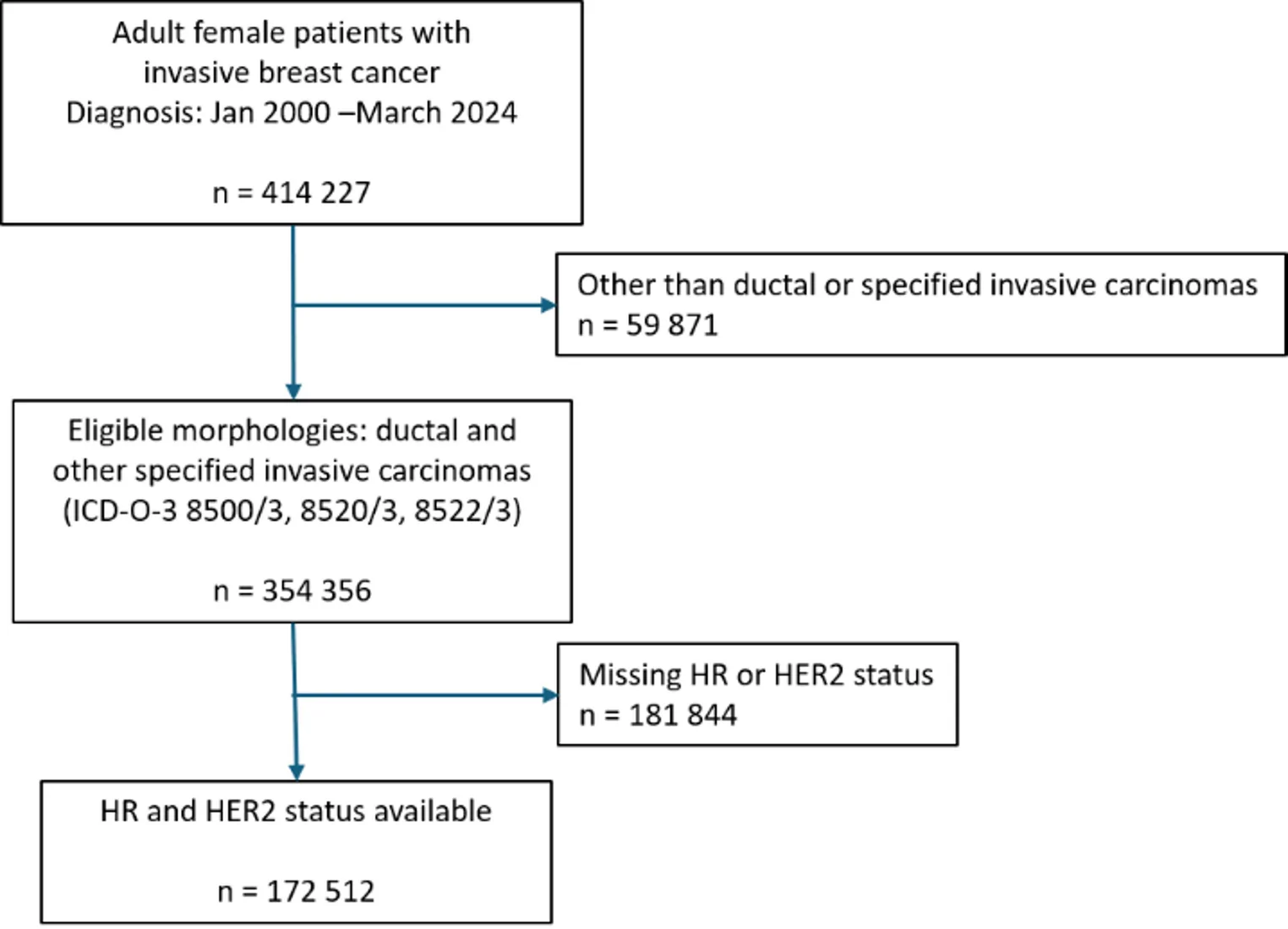
Flow-chart of study population selection

We identified 414 227 women aged ≥18 years with invasive breast cancer. After applying the inclusion criteria (ICD-O-3 8500/3, 8520/3, 8522/3) and exclusion of cases without available HR and HER2 status, the final analytical cohort comprised 172 512 patients (Fig. 1).

The cohort included 40 003 patients diagnosed in 2000–2009, 53 107 in 2010–2016, and 79 402 in 2017–2024. The proportion of patients diagnosed with synchronous metastases remained relatively stable at 6–7% across calendar periods. In contrast, the proportion of recorded metachronous metastases decreased from approximately 16% in earlier diagnosis periods to 5% in the most recent period, reflecting the shorter available follow-up in more recently diagnosed patients.

Baseline characteristics of the study population according to metastatic status are shown in Table 1. Patients with synchronous metastases were older at diagnosis (median age 70 years) compared to those with metachronous metastases (61 years) and those without metastases (64 years). In addition, the group of patients with synchronous metastases showed larger tumors and lymph node involvement more frequently.

**Table 1:** Baseline characteristics of study population

	Synchronous (N=11 290)	Metachronous (N=15 646)	No metastases (N=145 576)	Total (N=172 512)
Age at diagnosis of primary tumor				
Mean (SD)	68.0 (13.6)	61.0 (14.1)	63.7 (13.8)	63.7 (13.9)
Median [IQR]	70 [58, 79]	61 [50, 72]	64 [53, 74]	64 [53, 75]
TNM-T				
T1	1293 (12%)	3791 (24%)	75,142 (52%)	80,226 (46%)
T2	3452 (31%)	7773 (50%)	51,894 (36%)	63,119 (37%)
T3	1187 (10%)	1703 (11%)	6231 (4%)	9121 (5%)
T4	4222 (37%)	1696 (11%)	5779 (4%)	11,697 (7%)
TX or unknown	1136 (10%)	683 (4%)	6530 (4%)	8349 (5%)
TNM-N				
N0	2079 (18%)	5470 (35%)	96,179 (66%)	103,728 (60%)
N1	4513 (40%)	5612 (36%)	32,405 (22%)	42,530 (25%)
N2	1553 (14%)	1965 (13%)	6328 (4%)	9846 (6%)
N3	1671 (15%)	1795 (11%)	3111 (2%)	6577 (4%)
NX or unknown	1474 (13%)	804 (5%)	7553 (5%)	9831 (6%)
Subtypes				
HR+/HER2-	7415 (66%)	9957 (64%)	108,301 (74%)	125,673 (73%)
HR-/HER2-	1321 (12%)	2871 (18%)	15,075 (10%)	19,267 (11%)
HR+/HER2+	1689 (15%)	1862 (12%)	16,252 (11%)	19,803 (12%)
HR-/HER2+	865 (8%)	956 (6%)	5948 (4%)	7769 (5%)
Grading				
G1	675 (6%)	847 (5%)	25,508 (18%)	27,030 (16%)
G2	6119 (54%)	7927 (51%)	80,736 (56%)	94,782 (55%)
G3	4007 (36%)	6442 (41%)	37,124 (26%)	47,573 (28%)
GX	489 (4%)	430 (3%)	2208 (2%)	3127 (2%)
early surgery (within 6 months after diagnosis and before metastasis or within 3 months for synchronous metastasis)				
Breast conserving therapy/Mastectomy	2661 (24%)	7491 (48%)	95,767 (66%)	105,919 (61%)
Other	1456 (13%)	4801 (31%)	19, 241 (13%)	25,498 (15%)
No surgery / after metastasis	7173 (63%)	3354 (21%)	30,568 (21%)	41,095 (24%)
chemotherapy				
Yes	3872 (34%)	9730 (62%)	52, 744 (36%)	66,346 (38%)
endocrine therapy (HR+ patients only, within 6 months)				
Yes (from HR +)	4740 (52%)	4701 (40%)	55,344 (44%)	64, 785 (45%)
Radiotherapy				
Yes	3117 (28%)	5801 (37%)	64,371 (44%)	73,289 (42%)

With respect to molecular subtypes, HR+/HER2− tumors represented the most common subtype across all groups. The distribution of molecular subtypes differed between patients with synchronous and metachronous metastases.

### Synchronous metastases

**Fig. 2:**
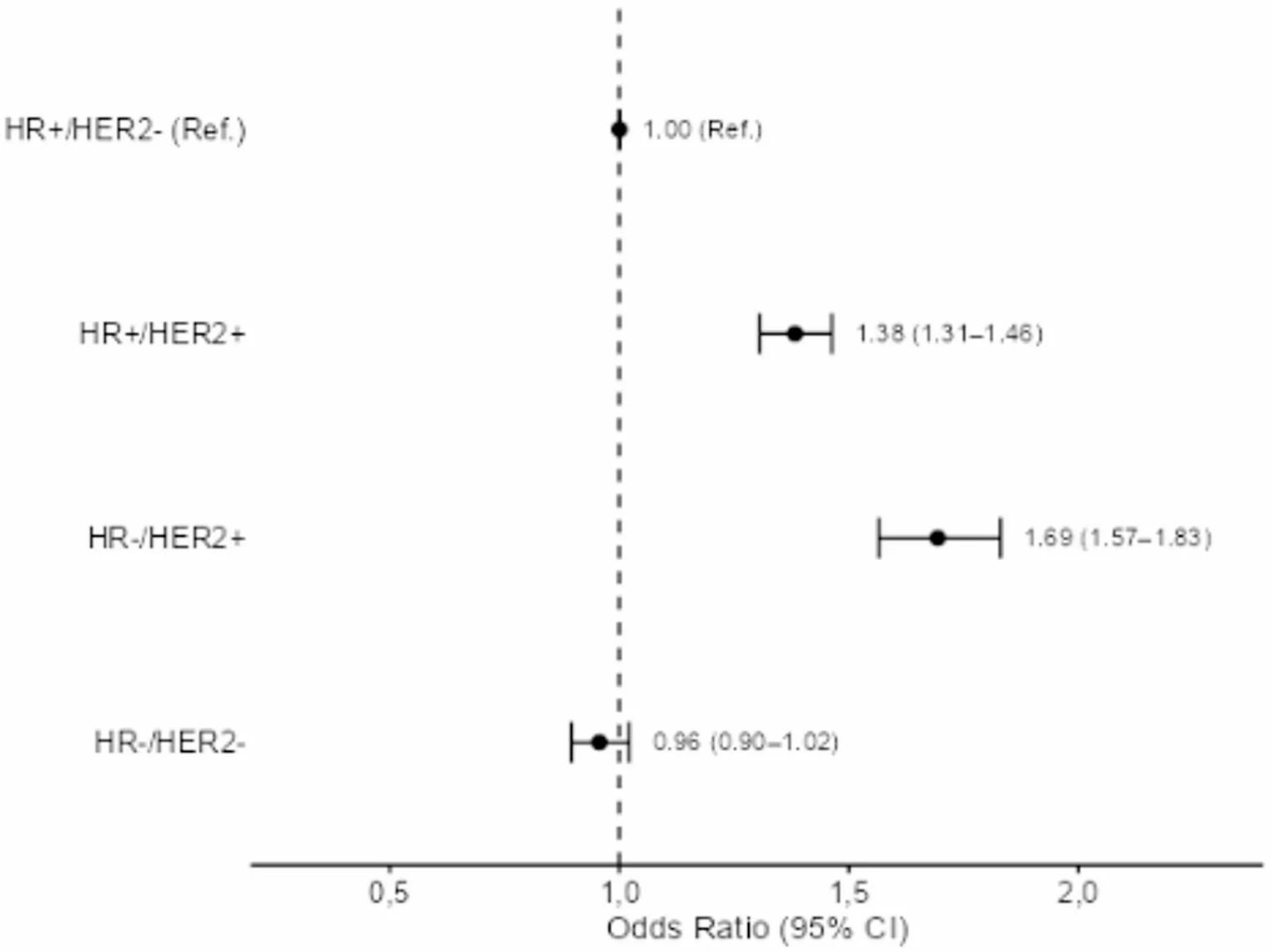
Association between molecular subtype and synchronous metastases at diagnosis. Odds ratios from binary logistic regression adjusted for age group, histological grade, calendar period, and registry source

After adjustment for age, histological grade, calendar period, and registry source, the odds of synchronous metastases differed according to molecular subtype (Fig. 2). Compared to HR+/HER2− tumors, patients with HR+/HER2+ tumors had 40% higher odds of presenting with synchronous metastases (adjusted OR [aOR] 1.38, 95% CI 1.31–1.46). Moreover, HR−/HER2+ tumors showed the strongest association with synchronous metastases (aOR 1.69, 95% CI 1.57–1.83). In contrast, HR−/HER2− tumors were not associated with an increased likelihood of synchronous metastases (aOR 0.96, 95% CI 0.90–1.02).

Older age and higher histological grade were independently associated with synchronous metastases. Compared with patients aged 18–49 years, those aged 70 years or older had substantially higher odds of synchronous metastatic presentation (aOR 2.44, 95% CI 2.29–2.60). Similarly, higher tumor grade was associated with increased odds, with grade 3 tumors showing the strongest association compared with grade 1 tumors (aOR 3.40, 95% CI 3.12–3.71).

### Metachronous metastases

**Fig. 3:**
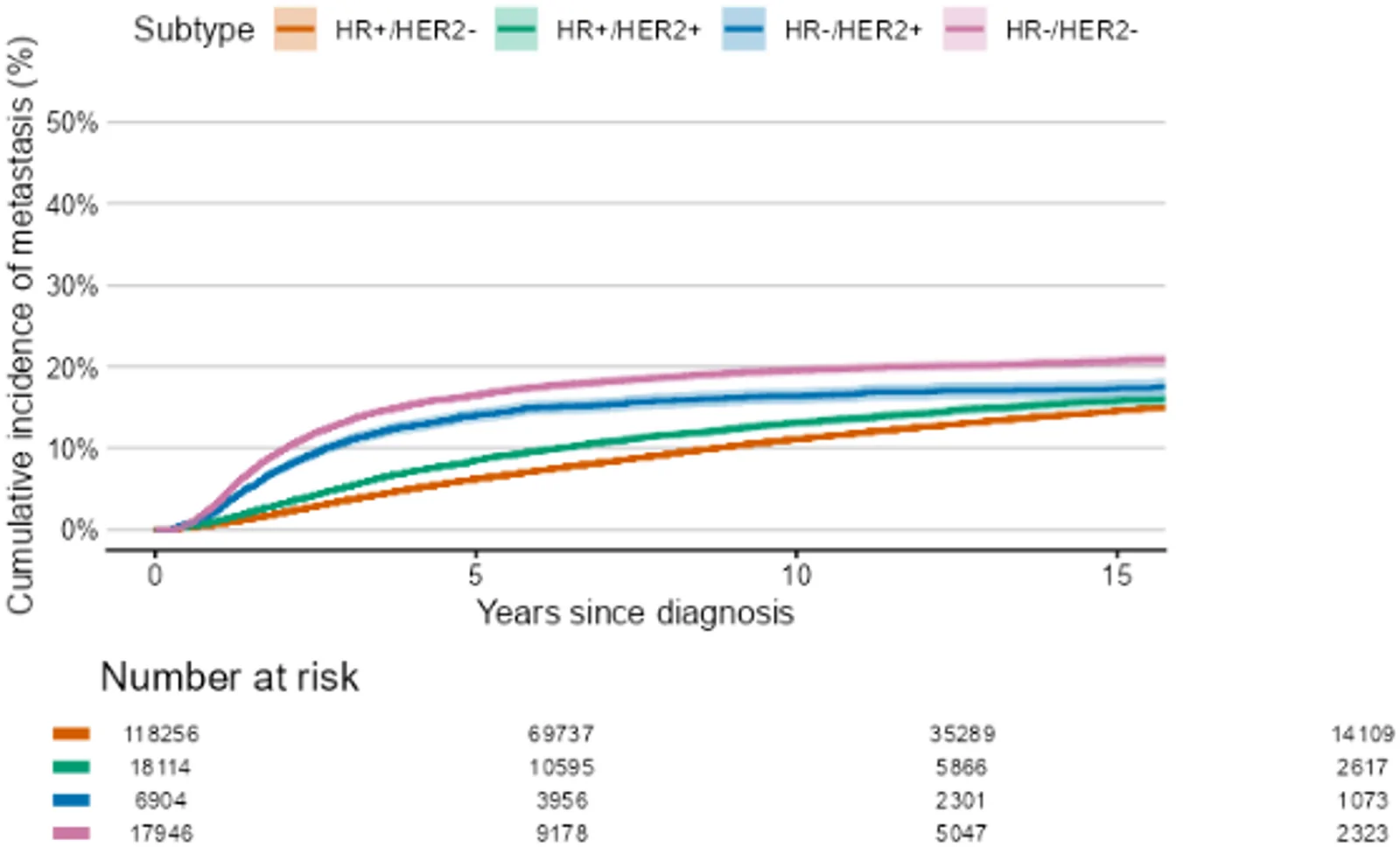
Kaplan–Meier estimates of the cumulative incidence of metachronous metastasis according to molecular subtype among patients without synchronous metastatic disease at diagnosis (1 − survival probability). For graphical presentation, the x-axis was limited to 15 years of follow-up and the y-axis to a cumulative incidence of 50%

Among patients without synchronous metastases at diagnosis (n = 161 222), the mean and median follow-up durations were 7.5, and 6.2 years, respectively. The cumulative incidence of metachronous metastases differed substantially according to molecular subtype (Fig. 3). Patients with HR−/HER2− tumors showed the highest cumulative incidence throughout the follow-up period, whereas HR+/HER2− tumors showed the lowest risk. At 5 years, the cumulative incidence of metachronous metastasis was 17% for HR−/HER2− tumors, compared to 14% for HR−/HER2+, 9% for HR+/HER2+, and 6% for HR+/HER2− tumors. By 10 years, the cumulative incidence had increased to 20%, 16%, 13%, and 11%.

Across subtypes, cumulative incidence increased substantially between 5 and 10 years after diagnosis. Beyond 10 years, further increases were comparatively small and were mainly observed among HR+ subtypes, whereas the cumulative incidence among HR- tumors showed little additional increase.

These subtype-specific differences were accompanied by differences in age at metastatic presentation. Among patients who developed metachronous metastases, those with HR+/HER2− tumors were older at metastatic diagnosis (median 68 years), whereas patients with HR−/HER2− tumors were younger (median 62 years), with intermediate ages observed for HR+/HER2+ (65 years) and HR−/HER2+ tumors (63 years).

Among patients who developed metachronous metastases, median time to metastasis was shortest for HR−/HER2− (median 1.7 years) tumors and longest in HR+/HER2− tumors (median 3.6 years).

**Fig. 4:**
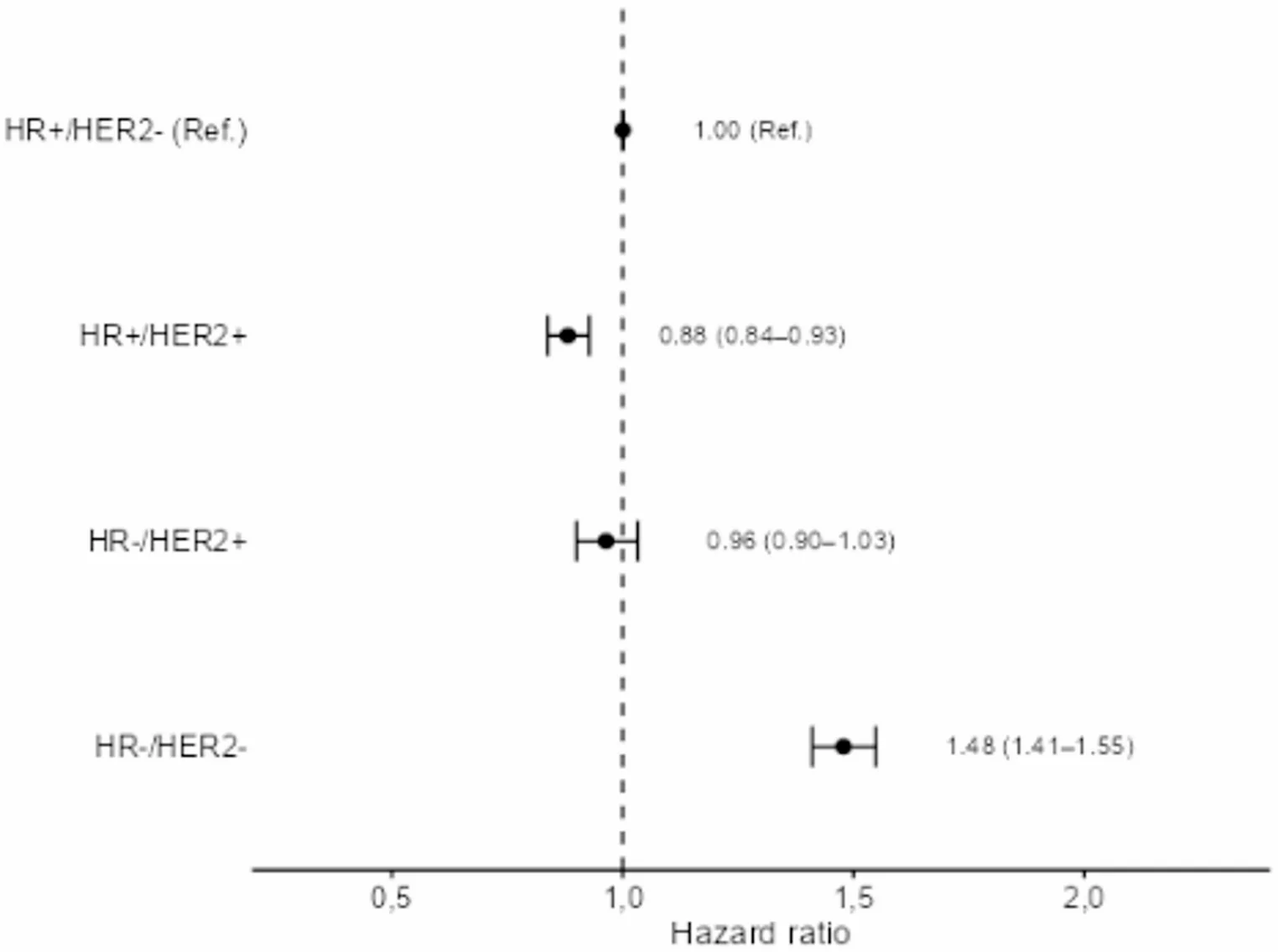
Risk of metachronous metastasis according to molecular subtype of breast cancer cases

In multivariable Cox regression analyses adjusted for age at primary breast cancer diagnosis, calendar period, T -status, N-status, histological grade, and registry source, molecular subtype remained independently associated with the risk of metachronous metastases (Fig. 4). Compared with HR+/HER2- tumors, patients with HR+/HER2+ tumors had a slightly lower hazard of metachronous metastasis (adjusted HR [aHR] 0.88, 95% CI 0.84 – 0.93), whereas no significant difference was observed for HR-/HER2+ tumors (aHR 0.96, CI 0.90–1.03). In contrast, HR−/HER2− tumors were associated with a markedly increased risk (aHR 1.48, CI 1.41–1.55).

Higher T stage, nodal involvement, and higher histological grade were all independently associated with an increased hazard of metachronous metastasis. In contrast, older age at diagnosis was associated with a slightly lower hazard after adjustment for tumor characteristics.

### Metastatic patterns

**Fig. 5:**
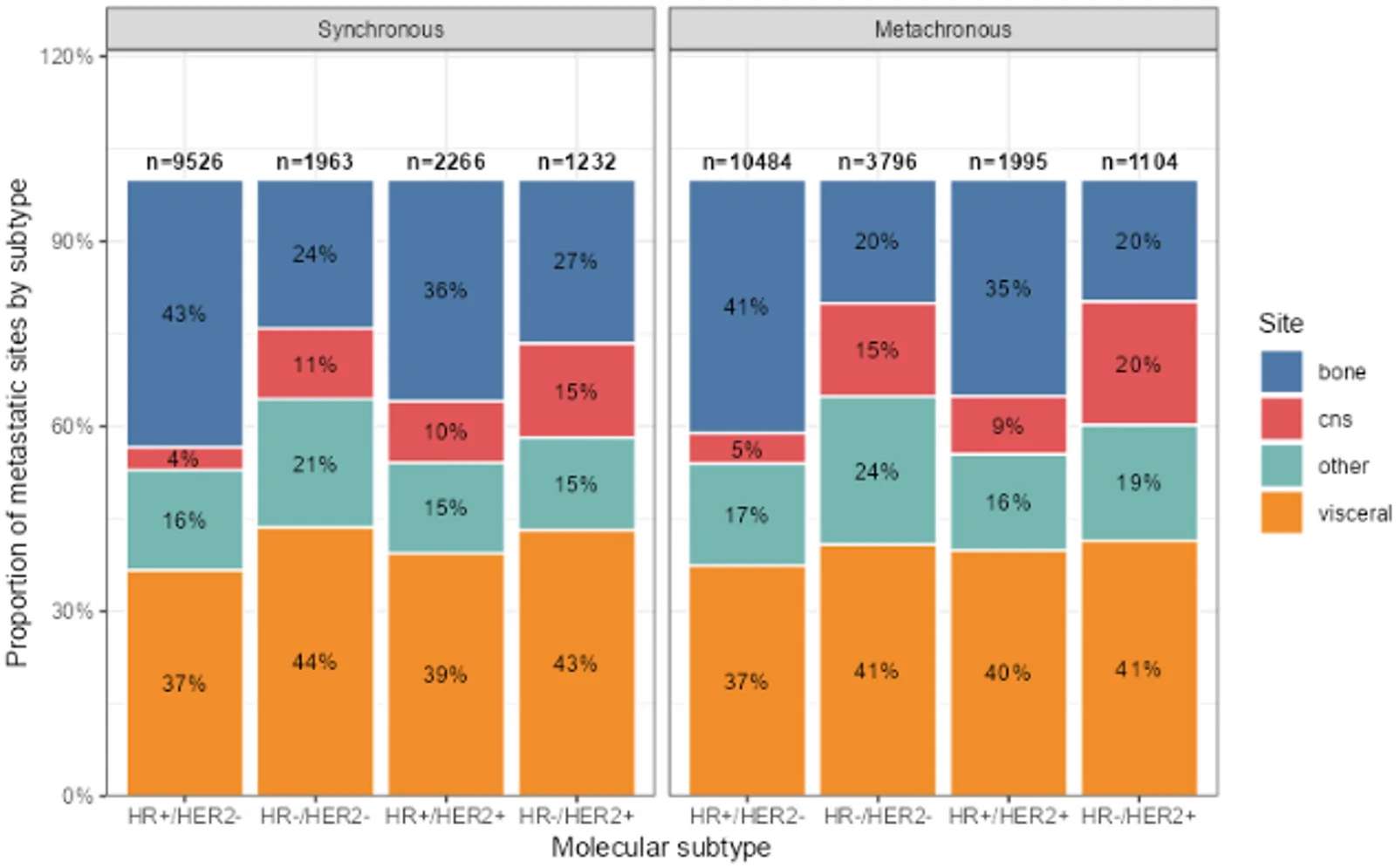
Distribution of metastatic sites of breast cancer patients stratified by molecular subtype and timing (synchronous vs metachronous)

Figure 5 illustrates the distribution of metastatic sites among patients with metastatic disease, stratified by molecular subtype and by synchronous versus metachronous presentation. Across all molecular subtypes, bone was a frequent metastatic site, although its relative contribution varied substantially between subtypes. HR+/HER2− tumors showed the highest proportion of bone involvement, consistent with a bone-predominant pattern of dissemination. In contrast, HR−/HER2− tumors demonstrated a lower proportion of bone metastases and a greater contribution of visceral and CNS metastases, suggesting a more aggressive and diverse metastatic pattern.

HER2-positive tumors showed an intermediate distribution, with a relatively balanced contribution of bone and visceral sites. Visceral metastases represented a substantial proportion of metastatic sites across subtypes, with the highest proportions observed among HER2-positive and HR−/HER2− tumors. CNS involvement was uncommon overall but appeared relatively enriched in HR−negative tumors, particularly HR−/HER2− disease.

Comparison of metastatic timings showed that differences between synchronous and metachronous presentations were less pronounced than subtype-associated differences.

Overall, these findings indicate that molecular subtypes influence not only the likelihood and timing of metastatic disease but also the anatomical pattern of dissemination. HR+/HER2− tumors displayed a predominantly bone-associated metastatic pattern, whereas HR−/HER2− tumors showed a greater propensity toward visceral and CNS involvement.

Taken together, these findings demonstrate that molecular subtypes are associated with multiple dimensions of metastatic disease. HER2-positive tumors were more likely to present with synchronous metastases, whereas HR−/HER2− tumors exhibited the highest risk of developing metachronous metastases during follow-up. Furthermore, metastatic dissemination patterns differed according to subtype, with bone-predominant spread in HR+/HER2− disease and greater visceral and CNS involvement in HR−/HER2− tumors. These results suggest that tumor biology influences not only the occurrence of metastatic disease but also its timing and anatomical distribution.

## Discussion

In this large population-based cohort of women with invasive breast cancer from German regional cancer registries, molecular subtypes were strongly associated with both the timing and anatomical distribution of metastatic disease. HER2-positive tumors were more likely to present with synchronous metastases, whereas TNBC (HR-/HER2-) showed the highest risk of metachronous metastases. Metastatic patterns also differed substantially by subtype, with HR+/HER2- tumors predominantly developing bone metastases and TNBC more frequently involving visceral organs and the CNS. By jointly evaluating synchronous, metachronous metastases and organ-specific dissemination within one large population-based cohort, our study provides a comprehensive picture of subtype-specific metastatic behavior. The distinct subtype distributions observed for synchronous and metachronous metastases are consistent with the possibility that these entities reflect, at least in part, different biological processes rather than merely different time points of the same disease process.

### Temporal patterns according to subtypes

Our findings are consistent with the current understanding of breast cancer biology, which recognizes molecular subtype as a major determinant of prognosis and metastatic behavior in breast cancer (Dent et al. 2007; Kennecke et al. 2010; Pan et al. 2017). HER2-positive tumors have been associated with an increased risk of metastatic presentation at diagnosis, reflecting their aggressive biology and high metastatic potential, whereas TNBC is characterized by a higher risk of early recurrence after primary treatment (Dent et al. 2007; Kennecke et al. 2010). These observations likely reflect the distinct biological characteristics of HER2-positive and TNBC tumors, although clinical outcomes for HER2-positive disease have changed substantially following the introduction of effective HER2-targeted therapies (D. Slamon et al. 2011; D. J. Slamon et al. 2001).

Differences in tumor evolution, metastatic dissemination, response to systemic therapy, and tumor dormancy may contribute to variation in the timing of metastatic progression (Massagué and Obenauf 2016; Aguirre-Ghiso 2007; Phan and Croucher 2020). This concept is particularly relevant for HR-positive tumors, which are characterized by a persistent risk of late recurrence, potentially reflecting the reactivation of dormant disseminated tumor cells years or even decades after initial diagnosis(Bushnell et al. 2021).

The temporal pattern of metachronous metastasis differed markedly across subtypes. TNBC showed the highest risk during the early follow-up period, consistent with previous reports describing an aggressive clinical course with early recurrence peaks (Dent et al. 2007; Kennecke et al. 2010; Metzger-Filho et al. 2013; Pan et al. 2017). In contrast, HR-positive tumors demonstrated continued increases in cumulative metastatic incidence between five and ten years after diagnosis, emphasizing the importance of prolonged follow-up in this subgroup (Pan et al. 2017).

The proportion of synchronous metastases remained relatively stable across calendar periods, despite advances in early detection and the introduction of population-based mammography screening. This finding should be interpreted cautiously. Our cohort was not restricted to screening-eligible age groups, and the reported proportions were not age-standardized. Furthermore, the proportion of synchronous metastases represents the proportion of patients with documented de novo metastatic disease rather than an age-standardized incidence rate. Therefore, the stable proportion observed in our study does not necessarily contradict stage shifts toward earlier disease that have been reported in screening-eligible populations.

### *Metastasis location according to subtypes.*

Subtype-specific differences were also observed in metastatic sites. HR+/HER2- tumors predominantly developed bone metastases, whereas TNBC showed more frequent visceral and CNS involvement. These findings are consistent with previous clinical studies describing distinct patterns of metastatic spread according to molecular subtype, with HER2-positive and TNBC showing increased propensity for visceral and brain metastases compared with HR+/HER2- tumors (Chen et al. 2018; Xiao et al. 2018). This organotropism is thought to arise from interactions between tumor-intrinsic properties and the tissue-specific metastatic microenvironment. Tumor cells with distinct molecular characteristics may differ in their ability to disseminate, survive in the circulation, cross tissue barriers, and adapt to specific organs. Conversely, organ-specific niches provide distinct chemokine and growth-factor signals, extracellular matrix components, stromal cells, and immune environments that can promote or restrict the survival and outgrowth of disseminated tumor cells. Together, these reciprocal interactions between tumor cells and the pre-metastatic or metastatic microenvironment may contribute to the subtype-specific patterns of metastatic spread observed clinically (Massagué and Obenauf 2016).

### Strengths and limitations

A major strength of this study is the inclusion of more than 170,000 women diagnosed over a 24-year period using harmonized data from multiple German regional clinical cancer registries. The large population-based design reflects routine oncological care and enhances the external validity of our findings beyond selected clinical trial populations. In addition, the long observation period enabled simultaneous evaluation of synchronous metastatic presentation and long-term metachronous metastases while also including patients diagnosed in recent years who received contemporary systemic therapies.

Several limitations should be considered. The retrospective observational design limits causal interpretation, and residual confounding cannot be excluded. Metastatic events were identified through registry documentation and clinical reporting and may therefore be subject to incomplete ascertainment or misclassification. Completeness of recurrence ascertainment may have varied across calendar periods and participating registries, particularly in earlier years before comprehensive clinical cancer registration was established. Deaths without a previously documented metastasis were recorded as deaths but were not assumed to indicate metastatic disease. Thus, some metachronous metastases may have been under-ascertained, particularly in earlier registry periods, which should be considered when interpreting temporal patterns. Furthermore, detailed information on treatment adaptation during follow-up and systematic validation of metastatic events was not available. During the long study period, systemic treatment strategies evolved substantially, particularly with respect to HER2-targeted therapies, endocrine therapy, and CDK4/6 inhibitors. Although calendar periods were included in the analyses, residual confounding due to temporal changes in treatment cannot be excluded.

## Conclusion

To our knowledge, few population-based studies have simultaneously evaluated molecular subtypes in relation to synchronous metastatic presentation, metachronous metastases, and organ-specific dissemination within a single cohort. Our findings complement previous evidence from German cancer registry-based studies, further highlighting the value of population-based data for understanding breast cancer progression and treatment outcomes (Engel et al. 2023). Overall, our results demonstrate that molecular subtypes influence not only whether metastatic disease develops, but also when and where it occurs. These results support subtype-adapted surveillance strategies, including closer early follow-up for patients with TNBC and prolonged follow-up among patients with HR-positive tumors. Recognizing these subtype-specific patterns may facilitate more individualized surveillance strategies and improve our understanding of metastatic breast cancer progression.

## Data Availability

The datasets generated during and/or analyzed during the current study are available from the corresponding author on reasonable request, following permission of the cancer registries. Access is subject to applicable German regional cancer registry legislation.
